# IgE-Mediated and Non-IgE-Mediated Fish Allergy in Pediatric Age: A Holistic Approach—A Consensus by Diagnostic Commission of the Italian Society of Pediatric Allergy and Immunology

**DOI:** 10.3390/medicina59091651

**Published:** 2023-09-12

**Authors:** Carla Mastrorilli, Stefania Arasi, Simona Barni, Davide Caimmi, Fernanda Chiera, Pasquale Comberiati, Giulio Dinardo, Arianna Giannetti, Marco Gismondi, Serena Gracci, Francesco Paravati, Umberto Pelosi, Michele Miraglia Del Giudice, Roberto Bernardini, Luca Pecoraro

**Affiliations:** 1 Admission and Emergency Pediatric Medicine and Surgery Unit, University Hospital Consortium Corporation Polyclinic of Bari, Pediatric Hospital Giovanni XXIII, 70124 Bari, Italy; carla.mastrorilli@policlinico.ba.it (C.M.); marco.gism@gmail.com (M.G.); 2 Area of Translational Research in Pediatric Specialities, Allergy Unit, Bambino Gesù Children’s Hospital, IRCCS, 00165 Rome, Italy; stefania.arasi@opbg.net; 3 Allergic Unit, Department of Pediatric, Meyer Children’s Hospital, 50139 Florence, Italy; simonabarni@hotmail.com; 4 Allergy Unit, CHU de Montpellier, Université de Montpellier, 34295 Montpellier, France; davide.caimmi@gmail.com; 5 IDESP, UMR A11, Université de Montpellier, 34093 Montpellier, France; 6 Department of Pediatrics, San Giovanni di Dio Hospital, 88900 Crotone, Italy; fernandachiera@hotmail.it (F.C.); paravati.f@gmail.com (F.P.); 7 Section of Paediatrics, Department of Clinical and Experimental Medicine, University of Pisa, 56126 Pisa, Italy; pasquale.comberiati@gmail.com; 8 Department of Woman, Child and of General and Specialized Surgery, University of Campania “Luigi Vanvitelli”, 80138 Naples, Italy; dinardogiulio@gmail.com (G.D.); michele.miragliadelgiudice@unicampania.it (M.M.D.G.); 9 Pediatric Unit, IRCCS Azienda Ospedaliero-Universitaria di Bologna, 40138 Bologna, Italy; ariannagiannetti25@gmail.com; 10 Pediatrics and Neonatology Complex Unit, San Giuseppe Hospital, Azienda USL Toscana Centro, 50053 Empoli, Italy; serena.gracci@uslcentro.toscana.it; 11 Department of Pediatrics, University Hospital of Pisa, 56124 Pisa, Italy; 12 Pediatric Unit, Santa Barbara Hospital, 09016 Iglesias, Italy; umbertopelosi@gmail.com; 13 Pediatric Unit, Department of Surgical Sciences, Dentistry, Gynecology and Pediatrics, University of Verona, 37126 Verona, Italy; lucapecoraro88@gmail.com

**Keywords:** fish allergy, management, molecular diagnostics, pediatrics

## Abstract

Fish is one of the “big nine” foods triggering allergic reactions. For this reason, fish allergens must be accurately specified on food labels. Fish allergy affects less than 1% of the world population, but a higher prevalence is observed in pediatric cohorts, up to 7%. Parvalbumin is the main fish allergen found in the muscles. In childhood, sensitization to fish allergens occurs most frequently through the ingestion of fish, rarely transcutaneously or by inhalation. Fish allergy symptoms usually appear within two hours of the allergen contact. The diagnosis begins with the collection of the history. If it is suggestive of fish allergy, prick tests or the measurement of serum-specific IgE should be performed to confirm the suspicion. The oral food challenge is the gold standard for the diagnosis. It is not recommended in case of a severe allergic reaction. It is important to make a differential diagnosis with anisakiasis or scombroid poisoning, which have overlapping clinical features but differ in pathogenesis. Traditionally, managing fish allergy involves avoiding the triggering species (sometimes all bony fish species) and requires an action plan for accidental exposures. The present review will analyze IgE- and non-IgE-mediated fish allergy in children from epidemiology, pathogenesis to clinical features. Moreover, clinical management will be addressed with a particular focus on potential nutritional deficiencies.

## 1. Introduction

Fish represents one of the most common food allergens, particularly in countries with high consumption of the same (e.g., China, Japan, Portugal, Spain, and Scandinavian countries). Specifically, the increase in fish consumption since childhood due to its great nutritional value and benefits (high content of antioxidants and omega-3 fatty acids) has led more frequently to allergic reactions to this food. Therefore, fish is one of the “big nine” allergenic foods responsible for allergic reactions. Fish belongs to the European Union food labeling regulation (EU Regulation No. 1169/2011) as an essential food component and a potent allergenic source. Regardless of the content percentage, it must be listed as an ingredient specifically on the product’s label [1]. Globally, many fish species are traded, which varies according to local production sites and dietary habits in different countries. Although cod and salmon are the most important fish in Europe, other fish species are popular in Asia (e.g., carp). Allergens have been labelled in about 40 fish species. Still, a detailed analysis of allergenic molecules has been performed principally for fish commonly consumed in Europe, such as carp, cod, salmon, trout, and tuna. Clinically, fish can be involved in IgE- or non-IgE-mediated adverse food reactions. This article analyzes the main fish allergens and evaluates the evidence on epidemiology, diagnostic work-up, treatment, and prognosis of fish allergy in children. Finally, we will focus on potential nutritional deficiencies caused by an exclusion diet as a treatment for fish allergy. This will provide a significant understanding of fish allergy in children.

## 2. Allergenic Source

### 2.1. Taxonomic Classification

**Figure 1:**
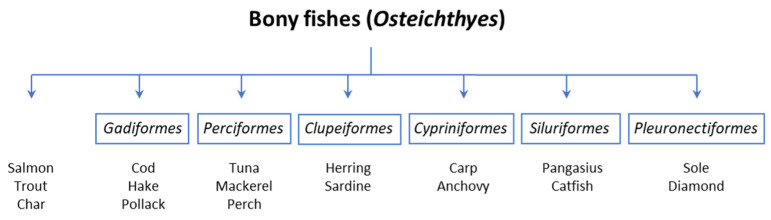
Main taxonomic orders belonging to the superclass of *Osteichthyes*.

The term “fish products” includes fish and seafood, as well as crustaceans and mollusks (e.g., prawns, crabs, lobsters, mussels, oysters, octopus, squid), classified into three Phyla: *Mollusca*, *Arthropoda*, and *Chordata*, each subdivided into various classes and species [2] (Table 1). Regarding the phylogenetic distance among fishes and these other organisms, it is not surprising that cross-reactivity between fish and shellfish has not been assessed [3]. Therefore, seafood will not be discussed in this article. *Chordata* includes finned fish and can be divided into bony fish, to which most edible species belong, and cartilaginous fish [4]. Despite the wide biodiversity among fish (more than 30,000 species have been reported), the most frequently consumed species are the members of the group Osteichthyes (bony fish) that belong to a limited number of orders: salmon (*Salmoniformes*), cod (*Gadiformes*), perch (*Perciformes*), herring (*Clupeiformes*), carp-like fish (*Cypriniformes*), catfish-like (*Siluriformes*), and flatfish (*Pleuronectiformes*) (Figure 1). Fish is eaten in several forms: cooked, fried, pickled, or raw. Food processing appears not to influence the allergenic potency of fish, but rather the allergenic content, which varies in different species [5]. In particular, the allergenic potency has been linked to peculiar protein characteristics, such as high stability toward food processing and digestion. The structural stability has been attributed to intrinsic ligand binding or intramolecular disulfide bonds, so that some food allergens combine protein aggregates. Fish allergens can be distinguished according to their location.

### 2.2. Fish Muscle

The significant allergenic activity lies in the fish muscle [1]. Parvalbumin is the main fish allergen and is very abundant in fish muscles. In a portion of 200 g of cod fillet, the consumer can ingest up to 0.5 g of parvalbumin per meal. Supplementary allergens present in fish muscles are enolase, aldolase, collagen, and tropomyosin [5].

### 2.3. Fish Roe

Even products (fish roe, caviar, skin, gelatin, and blood) derived from fish can have allergenic properties [18]. Typically, fish roe is consumed in its raw form. There are case reports citing caviar as responsible for allergic reactions. Vitellogenin has been identified as a major allergen of fish roe [19]. This protein and its metabolites account for almost all the total protein content of eggs. Recently, knowledge of fish roe allergens has improved, and the first recombinant molecules have been produced [18].

### 2.4. Fish Gelatin

Additionally, fish gelatin consists of hydrolyzed collagen made from fish skin and bones; conversely, isinglass is obtained from fish swim bladder and similarly comprises predominantly collagen [20]. These ingredients can be encompassed into foods (beverages, candies), drugs (capsules and gel coatings), or biologicals (vaccines, sublingual immunotherapy) [4,21]. Allergenicity seems to be related to collagen-like products (derived from fish skin, scales, and bones) [22] but could also be caused by contaminations of fish meat residues. Consumers are often unaware of fish-derived food ingredients, since they are exempt from the food labeling regulation. The food industry also uses fish blood (hemin or other blood proteins) as an additive during processing but appears to be a significant source of allergens just in the fish processing environment. Occupational asthma could be related to aerosolizing potential blood-derived allergens during fish processing. It has been recommended that a potential allergen may be serum albumin, but this is also because no other allergens have been confirmed so far [23].

## 3. Epidemiology

Fish is considered one of the most frequent foods that can cause anaphylactic reactions. Prevalence depends on the geographical area as it is generally higher in communities where fish is consumed in high quantities, such as China, Japan, Portugal, Spain, and Scandinavian countries. There is a lack of epidemiological studies on fish allergies presenting consistent fish sensitization data, and those published lead to specific prevalence rates in study design and variable methodology [24,25,26]. Less than 1% of the world’s population appears to be affected by a fish allergy. A higher percentage is observed in pediatric cohorts and countries with long seashores, elevated fish consumption, and regions characterized by fish processing industries (up to 3%) [27]. Given that less than 10% of patients with fish allergy have a resolution with age, this clinical condition is reported to be more frequent in adulthood, and the prevalence in children is less than 0.2% [5]. Epidemiological data on the prevalence of fish allergy are predominantly based on self-reported reactions, less frequently based on in vivo or in vitro diagnostic tests, and ultimately on oral challenge (Table 2).

### 3.1. Epidemiology of IgE-Mediated Fish Allergy: Self-Reported Data

Investigating parent-reported reactions, the highest prevalence was found in Finnish children aged 1–6 years, accounting for 5–7% [28,29]. Conversely, the lowest prevalence in children aged 0–2 years (0.0001%) is measured in Israel [30]. A significant prevalence has also been reported in the United Arab Emirates [31], where 2.8% of children aged 6 to 9 years have fish-related reactions (considering that the prevalence of food allergy was 8% in the same population). In Europe, the highest prevalence is registered in countries with traditional fish-focused diets, such as Spain or Scandinavian countries, especially Norway (3%), representing 17.8% of allergic reactions to foods [32]. Fish allergy was reported in 0.1% of US children’s food allergies according to a randomized cross-sectional survey, comparing 0% in children aged 0–5 years versus 0.2% in children aged 6–17 years [33]. Similarly, a latter American telephone survey showed a prevalence of fish allergy between 0.3% in children aged 0–2 years and 0.6% in children aged >11 years [34]. Fish allergy has a considerably high prevalence in Asian countries. A Singapore study of 227 children with a history of food allergy described fish as the causative allergen in 13% of cases. Interestingly, the first fish intake appears very early in Asian weaning, with an average age of only seven months [35]. The Philippines, Thailand, and Vietnam show prevalence rates of 2.29% [36], 1.1% [37], and 1.62% [38], respectively. Finally, the only available data on Africa can be deduced from a questionnaire-based study proposed in Ghana to a cohort of school-age children (5–16 years), displaying a prevalence rate of 0.3% [39].

### 3.2. Epidemiology of IgE-Mediated Fish Allergy: In Vivo or In Vitro Diagnostic Tests

These rates are lower when considering studies that included patients with fish allergy diagnosed by serum IgE measurements or percutaneous tests. In Europe, prevalence rates in pediatric cohorts are recorded as follows: 0.3% in Finland [40], 1.3% in the United Kingdom [41], and 0.7% in France [42]. An equally low prevalence rate of 0.21% is shown in Asia, specifically in China [43]. In contrast, a Spanish study [44] registered a frequency of documented allergic reactions to fish equal to 17.8% in a cohort of pediatric patients (clinical history, skin prick test, and positive serum IgE).

### 3.3. Epidemiology of IgE-Mediated Fish Allergy: In Vivo or In Vitro Diagnostic Tests

This prevalence decreases when considering oral challenge-diagnosed allergies, starting at 0% in Denmark [45], 0.0006% in the UK [46], 0.0002% in Turkey [47], and 0.2% in Iceland [48]. A meta-analysis confirmed the overall point prevalence of fish allergy assessed by oral challenge in Europe at 0.06% [49].

### 3.4. FPIES Induced by Fish: Epidemiological Data

Geographically, fish was the third lowest offending food in a population of 441 children with food protein-induced enterocolitis (FPIES) [34]. Conversely, fish was the first or second most implicated food triggering FPIES in Mediterranean countries, other than the most frequent trigger of solid food protein allergy [6,7,8,9,10,11,12,13,14,15,16,17,50].

## 4. Pathogenesis and Clinical Features

Pediatric fish allergy represents an adverse health reaction from a specific immune response that happens after the exposure to one or more specific fish species. According to immunological mechanisms, fish allergy can be divided into IgE-mediated if caused by immunoglobulin E antibodies against fish antigens and non-IgE-mediated if the immune response is played through cell-mediated mechanisms.

### 4.1. IgE-Mediated Pediatric Fish Allergy

IgE-mediated fish allergy is the most frequent and can occur through contact with the intestinal epithelium (ingestion), lung mucosa (inhalation), or skin. Sensitization and subsequent reactions occur most frequently after ingestion in the pediatric age. Nonetheless, they may also happen due to skin contact or inhaling aerosolized proteins spawned during cooking or processing. Fish antigens are absorbed about 10 min after ingestion, and even limited impairment of the denaturing effect of gastric acid (i.e., in patients using antacid drugs) may lead to partial digestion and intensified assimilation of allergens [51]. Specifically, a rise in gastric pH level from 2 to 3 causes a 10- to 30-fold increase in allergenicity for cod allergens [52]. In particular, in the case of anti-acid medication, there is a rapid uptake of fish allergens through the gastrointestinal tract in a cause-and-effect manner. The clinical report of IgE-mediated fish allergy is comparable to other food allergies. Single or multiple symptoms typically occur immediately or within two hours of exposure; conversely, delayed reactions up to eight hours after ingestion have been described [53]. Seafood products are one of the most common causes of life-threatening anaphylactic reactions. In particular, seafood allergies represent one of the most frequent causes of fatal food anaphylaxis [54]. Respiratory symptoms associated with oral allergy syndrome have been described more commonly than other food allergies [18].

### 4.2. Non-IgE-Mediated Pediatric Fish Allergy

Non-IgE-mediated immunological reactions to fish comprise eosinophilic esophagitis (EoE)/eosinophilic gastritis [55,56,57], FPIES and food protein-induced allergic proctocolitis (FPIAP) [11,58,59]. Although their underlying pathogenetic mechanism is nowadays poorly understood, cellular immunity is thought to drive an inflammatory response that can be expressed according to the affected segment of the gastrointestinal tract. In particular, a specific T cell response to causative antigens has been shown in FPIES. Moreover, it must be underlined that the management of EoE includes the empirical six-food elimination diet, which involves eliminating fish/shellfish and milk, eggs, wheat, nuts, and soybeans, which is generally recommended [10,60,61].

### 4.3. Clinical Clusters of IgE-Mediated Fish Allergy

The population allergic to fish can be divided into three clinical clusters: (1) polysensitized patients who react to all fish (“multiple fish allergy”); (2) oligo-sensitized patients reacting to several fish species; (3) monosensitized patients with “selective reactions” only to single fish species. Patients belonging to these clinical clusters vary according to IgE recognition profiles [62].

## 5. Diagnosis

**Figure 2:**
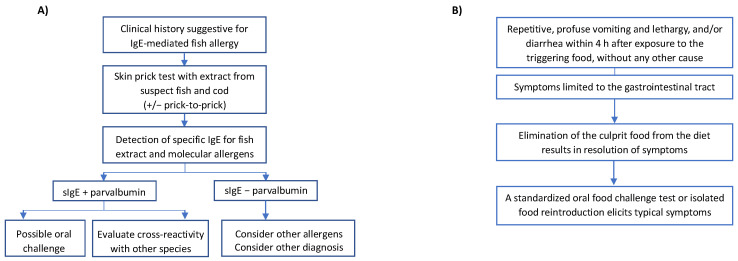
Diagnostic work-up of fish allergy in children. (**A**) Diagnostic examination in case of suggestive IgE-mediated allergy generally starts with skin prick tests and detection of serum sIgE for commercial extracts of the suspect fish and the cod as a screening test. Molecular allergology proposes the clinical relevance and helps to discriminate the clinical clusters of fish allergy. The gold standard diagnostic test remains the oral challenge with the suspect fish or with other species in order to evaluate possible cross-reactions. (**B**) The diagnosis of FPIES is based on clinical history according to symptoms and timing, other than exclusion of other causes (Powell’s criteria). The oral food challenge is required as gold standard for a diagnosis.

The diagnosis of fish allergy is principally based on clinical anamnestic criteria, percutaneous tests (prick test with fish extract or prick-by-prick method with triggering fish—raw and cooked), and evaluation of serum-specific IgE, followed (if necessary) by an oral food challenge (OFC) with the fish that elicited the reaction [63] (Figure 2A). A medical history suggestive of allergy remains the cornerstone of the diagnostic process. In particular, the diagnosis of FPIES is based on Powell’s clinical criteria (Figure 2B). Physician-supervised OFC is the gold standard for a diagnosis, and a periodic re-evaluation with OFC is recommended for monitoring the resolution [64]. Skin prick tests with commercial fish extract provide a rapid, non-invasive, inexpensive, and safe method of examining patients with a suggestive history of a reaction to fish. This test has a high negative predictive value (NPV), although the positive predictive value (PPV) is rarely greater than 50% [65]. It must be underlined that the results of skin tests can be influenced by the characteristics of commercial test reagents, as well as the technique/skills of the testing staff. Moreover, based on different companies, 26 commercial fish extracts were found as 10-fold varying in protein content, allergen concentration, and IgE reactivity by immunoblotting and mass spectrometry assays [66]. The fish species are tested using a raw extract or a prick-by-prick method. In light of cross-reactivity among fish species, cod prick testing has been suggested as a screening test for cross-reactivity (unless there is clinical reactivity to a specific fish) [5]. Sensitization, as assessed by serum IgE, correlates with clinical reactivity to predict fish allergy. In a US population, the level of IgE 20 kU/L for cod extract (ImmunoCAP, Thermo Fisher, Uppsala, Sweden) predicted cod allergy with 95% confidence [67]. More recently, an extensive oral challenge study registered that in patients with a suggestive clinical history, a serum-specific IgE value for cod extract >8.2 kU/L or for salmon extract >5 kU/L indicates cross-clinical reactivity [67]. These results, therefore, suggest that once the threshold values are exceeded, it should be recommended to avoid all fish species. Furthermore, a titer of specific IgE for cod >5 kU/L has been stated as a useful marker to define an unfavorable prognosis for the resolution of fish allergy [62]. In vitro diagnostic methods include the quantification of specific IgE for about 30 extracts of different fish species, as well as for allergenic molecules. The latter test (molecular diagnostics) is available with a singleplex method for particular molecules or in association with a standard panel of inhalant allergens and trophoallergens with a multiplex method. However, the number of allergens available in singleplex (ImmunoCAP) is limited to rGad c 1 and rCyp c 1 (major allergens of cod and carp) and therefore is not of much help if the patient is allergic to other fish species. Similarly, only rGad c 1 is available in the multiplex platform ISAC. Conversely, in the new multiplex platform ALEX2 (MacroArray Diagnostics, Wien, Austria), it is possible to quantify specific IgE for different fish species, as well as various fish allergens (cod, herring, mackerel, carp, salmon, swordfish, rays, tuna). Molecular diagnostics makes possible the distinction of patients monosensitized to a fish species from those with “multiple fish allergy”. A portion of patients affected by FPIES presents positive skin prick tests and/or serum fish-specific IgE. This peculiar form is called atypical FPIES and is usually persistent for longer [64]. For this reason, before reintroducing the culprit fish by OFC, it is suggested to perform diagnostic tests to shed light on a possible IgE sensitization. In this case, the food challenge should be carried out with the protocol of IgE-mediated food allergies [55]. The basophil activation test (BAT) represents a new diagnostic tool in food allergies that acts as a biomarker for predicting clinical reactivity and the severity of the reaction, aiming to decrease the need for OFC [68]. BAT uses flow cytometry to establish the expression of activation markers (e.g., CD 63, SD203c) on the surface of basophils through binding of the high-affinity IgE receptor (FcεRI) with IgE antibodies, which result from the stimulation with allergens or anti-IgE [69]. In a study conducted on 67 adolescent and adult patients, the significance of diagnostic tests (prick test, sIgE, IgG4, and BAT) was evaluated based on the double-blind placebo-controlled food challenge (DBPCFC) as the gold standard, evaluating the severity of allergic reactions to various foods (peanuts, hazelnuts, fish, shrimps, and sesame seeds) [70]. The findings showed that BAT may differentiate allergic patients from non-allergic patients, positively correlating with DBPCFC severity. Another recent study conducted on 51 Japanese children affected by fish allergy who underwent BAT using fish extracts from 15 various fish species [71] showed promising results for the five most frequently eaten fish species (salmon, mackerel, tuna, sea bream, and amberjack). While there are favorable results regarding its value in predicting clinical reactivity and the severity of allergic reactions, nowadays BAT is mostly performed for research, most likely due to technical complications (training of health care professionals and continuous quality assurance) and high cost.

## 6. Fish Allergens and Cross-Reactivity

The current WHO/IUIS database search shows 40 fish allergens, while the Allergen Online (www.allergenonline.org, version 22, accessed on 25 May 2023) includes 83 items in known sequence (Table 3). Of these, 16 and 40, respectively, belong to the parvalbumin family. Additional allergens are enolase (*n* = 5), aldolase (*n* = 4), tropomyosin (*n* = 3), salmon roe vitellogenin (*n* = 1), and others (*n* = 11). The main dominant allergen in fish muscle is undoubtedly parvalbumin, of which the first identified was Gad c 1, cod allergen [4]. Later studies were achieved with homologous proteins, such as Gad m1 from Atlantic cod, Cyp c 1 from common carp, and Sal s 1 from Atlantic salmon. Parvalbumins are small proteins (10–12 kDa) of the muscle with an important stability to the physicochemical effects of food processing. During fish preparation and cooking, these allergens can aerosolize and be inhaled, resulting in respiratory symptoms [23,72,73]. Due to specific features in their protein structure, these allergens can bind calcium with EF-hand domain structure [70]. Parvalbumin levels change noticeably among different fish species [4], with carp and herring containing about 100 times higher points than mackerel and tuna. Most fish-allergic patients show specific IgE to these allergens. Parvalbumin epitopes are highly conserved and may explain IgE and clinical cross-reactivity among various fish species. Parvalbumins differ in two molecular subtypes: alpha- and beta-lineage parvalbumins. Frequent fish allergens are beta-parvalbumins. In contrast, alpha-parvalbumins, such as those found in rays and sharks, show less cross-reactivity with beta-homologs. Parvalbumins have been identified as fish “panallergen”. Sensitization rates for beta-parvalbumins are based on allergen characterization studies, concluding for 90–95% IgE sensitization for these muscle proteins [1,65,74]. The prevalence of IgE sensitization to parvalbumin was shown to be lower than presumed for a long time. Depending on the study cohort, the sensitization rate to this important allergen could vary between 70% and 95% (Table 3). In addition, other fish allergens have been identified, such as 50 kDa-enolase and 40 kDa-aldolase from cod, salmon, tuna, and lately, Cyp c 2 from carp and Pan h 2 and Pan h 3 from catfish [74,75]. These enzymes with glycolytic effect are vastly expressed in fish muscles. Their strength as food allergens has yet to be identified since they are less stable than parvalbumins. Nevertheless, several fish-allergic patients appear to be sensitized to these allergens. In vitro, cross-reactivity arises among cod, salmon, and tuna homologs. Beyond parvalbumin, a polyclonal immune response to several fish allergens compares with clinical reactivity, as displayed for cod, salmon, and catfish [63]. A study showed that fish-allergic patients sensitized to cod parvalbumin can show specific IgE to enolase (81%) and aldolase (58%) [74]. The relevance of this co-sensitization is not clinically elucidated. Conversely, some parvalbumin-negative patients appear to show IgE antibodies against fish enolase (47%) and aldolase (41%), which are relatively related to species-specific allergies to fish [75]. Limited data have been reported delineating how many patients can be classified in every suggested clinical cluster. Furthermore, it must be considered that a geographical and temporal gradient could be appropriate to the prevalence of the collected data. Other relevant fish muscle allergens are, e.g., tropomyosins, creatine kinase, triosephosphate isomerase (illustrated in salmon and catfish), pyruvate kinase, lactate dehydrogenase, glucose-6-phosphate dehydrogenase, and glyceraldehyde-3-dehydrogenase (described in catfish) [75]. Further studies are awaited to confirm the clinical relevance of those allergens in a diagnostic panel. Fish gelatin is a heterogeneous product found by extracting acidic acid from collagen followed by chemical hydrolysis. Collagen comprises three single polypeptide chains corresponding to alpha subunits (α1, α2; each 110 kDa) and one beta subunit (210 kDa). These chains form a right-handed strait that winds up to arrange a rod-shaped triple helix. According to the molecular weight of the components, gelatin is available at different degrees of hydrolysis. Anaphylaxis due to fish gelatin allergy has been described in some case reports [76]. Fish gelatin varies significantly in its amino acid composition from its counterpart derived from mammals. Therefore, it is likely that there is no cross-reactivity between these products. Recently, the allergenic potency of fish collagen has been verified in various fish species, such as salmon, barramundi, and catfish, confirming previous reports [21,77]. Fish gelatin and collagen could be utilized as additives or processing aids in drugs, vaccines, and food products commonly thought to be fish-free and could thus be thought of as hidden allergens. The allergens in fish roe, known as caviar, differ from those in fish meat and skin. Patients with caviar allergies frequently tolerate fish meat, in contrast. Vitellogenins are glycolipoproteins belonging to the family of lipid transport proteins (LTP) with a high-molecular-weight (>150 kDa). Studies on salmonid roe allergens identified a 35 kDa vitellogenin fragment consisting of two partially identical subunits (18 and 16 kDa) referred to Onc k 5 [1]. Cross-reactivity was tested for egg allergens of different fish species using IgE and skin tests. Nevertheless, no cross-reactivity with chicken yolk homologs was found.

## 7. Differential Diagnosis

The ingestion of fish can lead to adverse reactions which may mimic an allergy but are not triggered by an immunological mechanism. The main non-immunological adverse reactions are listed below.

- Anisakiasis: Infestation by the parasite Anisakis (Nematode), with clinical manifestations mainly in the gastrointestinal tract. Requires ingestion of live parasites. Then, it is contracted only after consuming raw, undercooked, or pickled fish [78]. Within a few hours from ingestion, gastrointestinal symptoms occur (abdominal pain, vomiting, malnutrition). A severe eosinophilic granulomatous response can appear if larvae pass into the bowel. Diagnosis needs a gastroscopic examination by visualization of larvae which must be removed or biopsy with tissue histopathologic examination.
- Allergic reaction to Anisakis: IgE-mediated reaction to Anisakis due to sensitization of nematode proteins, which infest various fish species. The clinical presentation is not distinguishable from a fish allergy, but IgE is not directed against the fish but against the parasite proteins. Anisakis-specific prick tests and serum-specific IgE have been identified [79,80].
- Scombroid poisoning: The most common cause of toxicosis caused by seafood products worldwide. It is due to the ingestion of poorly preserved fish (most frequently red meat fish such as *Scombroidae* and *Scomberesocidae*, including mackerel, bonito, albacore, and tuna), in which the excessive bacterial growth allows the conversion of histidine into histamine. Clinical symptoms mimic allergic reactions in rapid onset (about 30 min after ingestion) and objectivity (e.g., urticaria, oral allergy syndrome, nausea and vomiting, and, in rare cases, anaphylaxis). Patients who habitually have no history of fish allergy may report oral tingling sensation, rash spreading to the face and trunk from top to bottom, and a metallic taste upon ingesting the culprit fish. Generally, the same symptoms and signs are reported by other diners who consume the same food [81,82]. Symptoms usually resolve within 24 h. Laboratory tests of dosing histamine levels from fish and patient’s plasma can help defining the diagnosis.
- Toxic Algae Poisoning: Adverse reaction caused by fish contaminated by toxic algae consumed by themselves. Clinical reactions are various and depend on the toxin involved: e.g., Ciguatera poisoning, due to the ciguatoxin, which is more commonly found in tropical fish (grouper, eel, mackerel), can present with skin (urticaria), gastrointestinal (nausea, vomiting), neurological (blurred vision, paresthesia, ataxia, convulsions), and cardiovascular symptoms (bradycardia/tachycardia, hypotension/hypertension, conduction block) [83,84].
- Bacterial/viral contamination: Poisoning caused by eating fish farmed or harvested from contaminated waters (e.g., by hepatitis A virus, *Shigella* spp., *Salmonella* spp.). It can mainly trigger gastrointestinal clinical manifestations several hours after ingestion, usually accompanied by fever [3].
- Seafood intolerance: Adverse reaction due to vasoactive biogenic amines in fish (histamine and tyramine), remarkably, if it is canned or pickled fish, or due to fish autolysis [85,86,87]. It usually presents with severe headache. It involves histamine as scombroid poisoning; however, clinical characteristics differ.

The characteristics of symptoms, the onset of reactions, and the lack of IgE sensitization to triggering fish are the main set of differences between fish allergy and non-immunological reactions to fish.

## 8. Clinical Management

The treatment of fish allergy is directed towards avoiding fish suspected of causing an allergic reaction and all other fish species, as well as prompt recognition and treatment of acute allergic reactions, including allergic reactions following inhalation of cooking vapors for the IgE-mediated forms [26]. There are two reasons for this event: firstly, the possible cross-reactivity among the different parvalbumins in the IgE-mediated forms; secondly, the difficulty of distinguishing some fish species from each other at the time of intake. Furthermore, since parvalbumin is a thermostable protein, raw and cooked fish should be avoided [26]. The problem of cross-reactions for IgE-mediated allergies is significant: they exist not only among different species of fish, but in rare cases, also with crustaceans, chicken, and crocodile meat [26]. Both the patient affected by IgE-mediated allergy to fish and non-IgE-mediated allergy can tolerate some species of fish, which can be reintroduced into the diet of a child allergic to a specific type of fish. Moreover, its tolerance must be verified using OFC setting [88]. In pediatric age, the diagnosis of fish allergy is common before the age of 2, often coinciding with the first intake of a fish species in the diet. In such cases, it is necessary to rigorously exclude fish from the diet at home and follow a specific diagnostic procedure for a progressive reintroduction of some fish species into the diet, starting with those with the most significant possibility of tolerance. In any case, until tolerance to a specific fish species is confirmed, the diet must avoid its intake.

### 8.1. Clinical Management of IgE-Mediated Fish Allergy

The positivity of specific IgE for parvalbumin does not always succeed in discriminating patients with allergy to a single fish species from those with multiple fish allergies, as they are often cross-reactive between homologs, not necessarily implying a clinical reactivity. An exception has been described for the subgroup of patients with monosensitization to salmonids (Salmoniformes), as there is a species-specific epitope of salmon/trout parvalbumin (Sal s 1+, Onc m 1+, Cyp c 1− Gad m 1−, Thu a 1−) [12]. Furthermore, it was recently described that cartilaginous fish could be tolerated by patients sensitized to bony fish due to low cross-reactivity among parvalbumins, which are only poorly correlated [89]. In the context of IgE-mediated forms, the choice of the first fish species to be reintroduced into the diet of a child suffering from multiple fish allergy falls on tuna and swordfish [90]. Particularly, these species consist mainly of red muscle, consequently comprehending a smaller amount of parvalbumin contained in the fish’s white muscle [90]. Moreover, tuna shares this characteristic with swordfish, which, however, does not possess the characteristic of being able to be subjected to processing and canning, with the result of involving a conformational change in the parvalbumin itself, making it less allergenic [91]. For this reason, canned tuna could be considered the first fish species to be tested in the reintroduction of fish into the diet of an allergic child, even if this occurrence has been demonstrated only in IgE-mediated allergy [92].

### 8.2. Clinical Management of Non-IgE-Mediated Fish Allergy

Non–IgE-mediated fish allergy is mainly related to FPIES [93]. As for IgE-mediated fish allergy, observational studies in fish-FPIES showed a certain tolerance to alternative species; tolerance to some fish different from the causative one was demonstrated. Moreover, swordfish and canned tuna were the best-tolerated fish, and all children who first received an allergic reaction to an alternative fish tolerated it. Furthermore, all types of fish must be avoided from diagnosis until the time the tolerance of an alternative fish species is demonstrated. Children affected by FPIES induced by fish should be tested with an alternative type of fish to avoid unnecessarily restricted diets [94]. Therefore, the standard care for both IgE- and non-IgE-mediated fish allergy is represented by food avoidance and rescue medication (e.g., adrenaline, corticosteroids, antihistamines) in case of accidental exposure to fish. The reintroduction of some fish species in the diet of the child suffering from fish allergy would assume an important role both on a nutritional level, ensuring the introduction of all the fish nutrients in the child’s diet, including, above all, omega-3, and the point of view of the quality of life of the child and his family, avoiding a restrictive diet. Finally, scientific research toward the definition of oral fish immunotherapy is still developing, as it happens in some specialized milk, egg, and peanut centers. Limited data have been published on fish oral immunotherapy using boiled or lyophilized fish. However, the efficacy of immunotherapy is not robust yet in desensitization, and a challenge can be the daily consumption of the triggering fish. Moreover, in contrast with milk and egg, fish is usually easy to avoid in the diet.

## 9. Nutritional Considerations

The exclusion diet from all fish species must be accompanied by the intake of foods with an excellent nutritional profile, aimed at leading to a healthy and balanced diet [95]. Fish is known to have a significant nutritional value: it is rich in vitamins of group B, D, and A, iodine, and omega-3. On the one hand, vitamins are commonly found in the diet taken from other animals, and iodine is added to table salt. Some examples are certain types of meat, legumes, vegetables, egg and dietary products [95]. On the other hand, omega-3 is present in a few other non-fish foods, such as seed oils and walnuts [95,96]. “Omega-3 fatty acids” refers to a large group of long-chain essential fatty acids and very long-chain polyunsaturated fatty acids. They are ingested through the diet and not synthesized endogenously. Omega-3 is incorporated into cell membranes and plays a key role in regulating inflammatory processes, specifically in growth both in pre-and postnatal cognitive development. Many authors recommend omega-3 supplementation in children suffering from fish allergy [95]. This recommendation originates from observational studies that reported that children affected by autism spectrum disorders, ADHD, and psychosis are affected by a blood deficiency of omega-3 fatty acids. Omega-3 fatty acids and omega-6 fatty acids and arachidonic acid exert different functions in neurotransmission, neurogenesis, and protection against oxidative stress. A review conducted by Agostoni et al. investigated the effects of omega-3 supplementation in these diseases, demonstrating weak evidence regarding their beneficial role as supportive therapy in neuropsychiatric disorders [96]. Concerning omega-3 supplementation in healthy subjects, the evidence is conflicting. A review conducted by the “Agency for healthcare research and Quality” demonstrated how the supplementation of omega-3, omega-6, and arachidonic acid in pregnant or lactating women or the use of formula milk enriched with these micronutrients does not bring a significant benefit both in pregnancy and infancy. Specifically, no significant effects of supplementation were found on gestational hypertension, peripartum depression, postnatal growth, autism spectrum, ADHD, learning disabilities, visual acuity pathologies, cognitive development, and prevention of allergic diseases and asthma [97]. In contrast, a meta-analysis by Papamichael et al. shows that the introduction of fish in the early stages of life (6–9 months) and the regular consumption of fish (at least once a week) reduces the subsequent incidence of acute wheezing episodes in children up to 4–5 years [98]. Supplementation of omega-3 fatty acids, if not adequately included in the diet by consuming flaxseed oil or walnuts, would be possible through supplements in patients with fish allergy. Although most manufacturers indicate on the label the likelihood of developing adverse reactions for patients allergic to fish, these supplements would be safe [99]. Further studies are needed to confirm these assessments. In conclusion, no studies deepened the possibility of administering an omega-3 supplementation in children with fish allergy or investigated the long-term outcome of children affected by IgE- and non-IgE-mediated fish allergy without a good nutritional plan to replace omega-3. It seems that a beneficial effect that is difficult to quantify could be attributed to supplementing omega-3 in these subjects. Moreover, no adverse effects related to an adequate intake of omega-3 in pediatric age have been reported in the literature. Given the potentially favorable risk/benefit ratio, omega-3 supplementation may therefore be recommended.

## 10. Prognosis and Natural History

### 10.1. Prognosis of IgE-Mediated Fish Allergy

Data on the natural history of IgE-mediated fish allergy are low; however, most evidence points to long-term clinical reactivity [100]. Previous evidence documented that about 80% of subjects showed a persistent picture of fish allergy ten years after diagnosis, while a telephone survey conducted in the USA found a resolution percentage of fish allergy of 3.5% [5,33]. A recent study conducted on 58 children showed, however, that a considerable proportion of children affected by fish allergy acquired tolerance often during adolescence, contrary to the theory according to which fish allergy persists until adulthood [60]. The age of onset of fish allergy ranges between 0.5 and 5 years, and 62% of children presented the first adverse reaction at the time of first intake of fish in the diet, a phenomenon related to a recall bias or sensitization from the dermal route, taking into account that 97% of the subjects had a history of atopic dermatitis. Moreover, 48% of subjects reacted only to one type of fish (generally cod) [60]. The percentage of tolerance increases with the age of the children, starting from 3.4% in children aged between 4 and 5 years to 11.8% in school-age subjects, up to 45.6% in adolescents. Predictive factors for the resolution of fish allergy are pre-challenge cod sIgE < 4.87 kUA/L, low relative values of recombinant Gad c 1, sardine SPT diameter < 6.5 mm, fish SPT diameter mix < 5 mm. The development of oral tolerance to fish mainly concerns subjects with less severe reactions and is associated with lower (or decreasing) values of specific IgE [60]. A total of 188 oral challenges to fish were performed in the study, of which 67 were with tuna, 46 with swordfish, 14 with cod in DBPCFC, and 38 with cod open challenge. Overall, 22% of the children developed a tolerance to cod during the follow-up period [60]. Similar results came from another retrospective study in which the median age of onset of symptoms is 24.2 months, and 74% of subjects acquired tolerance to at least one type of fish (most frequently tuna at 63%, followed by cod at 25% and salmon at 25%) at a mean age of 10.5 years [90]. In subjects who acquired tolerance to at least one type of fish, the mean value of rGad c 1 is significantly lower (5.1 kUA/L) than the initial value (16.8 kUA/L), as the mean value of sIgE for all fish species was tested (except tuna probably due to reduced parvalbumin content) and mean SPT diameter for hake (9.42 mm versus 3.79 mm) and salmon (7.8 mm versus 2.8 mm). The reduction in the diameter of the SPTs for the other types of fish did not reach statistically significant values, probably due to the small number of patients included in the study. These data demonstrate that the evaluation of sIgE for parvalbumin and SPT are relevant predictive factors in the follow-up of the subject with food allergy to fish, as they support the clinician in the timing of the oral challenge to verify the acquisition of tolerance and introduce new species of fish in the child’s diet [101]. Another recent study of 25 children with IgE-mediated fish allergies documented that 100% of the subjects could tolerate canned tuna [94]. Consumption of canned fish is associated with a reduction in SPT diameter in most patients and may favor the development of oral tolerance to fish [102].

### 10.2. Prognosis of Non-IgE-Mediated Fish Allergy

Acute FPIES is the most deeply investigated in literature since fish constitutes the main trigger among solid foods in some geographic areas (Italy, Spain, and Greece); also, the prognosis differs from FPIES caused by other foods [7,8,9,10,11,12,13,14,15,16,17,50,58,59]. A study set up in Spain on 17 children with FPIES documented that fish is the offending food in 80% of cases and hake is the most common trigger, while 41% have FPIES from multiple fish species. Tolerance was documented in only five subjects at a median age of about four years (while for the other solid foods considered, including banana, peach, rice, corn, and oats, the median age was three years). Tolerance to different fish species different from that triggering enterocolitis was documented in 8/13 subjects [46]. Similarly, the retrospective study by Miceli Sopo et al., conducted on 66 Italian children with FPIES, demonstrated that fish constitutes the second food-causing FPIES, preceded only by cow’s milk, and the median age of development of oral tolerance to fish is 4.4 years. A total of three/eight children with FPIES from one or more fish species tolerated other types of fish [44]. Furthermore, testing tolerance to a fish different from the one responsible for the adverse reaction is important because it could prevent excessive nutritional restrictions and favor oral tolerance induction [103]. Gonzalez-Delgado et al. reported a series of 16 children with fish-induced FPIES. In this study, all subjects presented symptoms after ingesting at least two different fish species and, in most cases, after introducing three types of fish (hake, whiting, and sole were the most commonly involved) [59]. In this cohort, only three subjects tolerated fish before the age of 5 years, and at least 50% of subjects presented persistent symptoms after the age of 6 years. In some cases, abdominal pain was reported after ten years [59]. Similarly, a study developed in Greece on a cohort of children with fish-induced FPIES confirmed that 52% of the subjects had not yet achieved tolerance by age 6 [11].

FPIES related to solid foods frequently have an older average resolution age than others. Regarding FPIES related to fish, the average resolution age seems to be 48–60 months, with a 75% probability of resolution at around 5 years [13,15]. An observational study by Sonsoles Infante et al. demonstrated that only two children outgrew their FPIES over 8 years, and fourteen of the seventy children (20%) did not overcome the disease after that age [94].

At the same time, 72.3% of the children in the study tolerated other fish, mostly canned tuna and swordfish. In addition, the risk factors for poor prognosis are not known. An observational study by Sonsoles Infante et al. demonstrated that the tolerant children developed symptoms when the first allergic reaction implied two different fish species. Nevertheless, the number of children who reacted with three or more fish species was similar in tolerant and non-tolerant groups. FPIES induced by fish has a worse prognosis than other types of food. The evolution of the disease is not related to the age of onset, the severity of the symptoms at the beginning, the number of fish the children had reacted to, or the coexistence of atopic diseases [94].

FPIAP induced by fish is not deeply investigated in the literature. It is known that it is rare, and its prevalence is 1%. The median age at diagnosis of FPIAP induced by fish is 2 months. Following a maternal Mediterranean diet during pregnancy and infancy protects against FPIAP. After diagnosis, breastfeeding should be continued, excluding the causative food from the maternal diet until FPIAP resolution. Tolerance is usually achieved by one year of age [104].

### 10.3. Prognosis and Natural History of IgE- and Non-IgE-Mediated Fish Allergy: Key Points

The prognosis of fish allergy varies depending on the type of allergic reaction (IgE- and non-IgE-mediated). IgE-mediated fish allergy is characterized by long-term clinical reactivity [100]. Specifically, the percentage of tolerance increases with the age of the children and seems related to the values of allergological tests and the severity of the first allergic reaction [60]. Non-IgE-mediated fish allergy is characterized by a low possibility of tolerance for the fish involved in the first allergic reaction in the case of FPIES [6,7,8,9,10,11,12,13,14,15,16,17,50]. Moreover, the possibility of tolerance of other fish species could be explored more deeply [94]. Regarding FPIAP, the prognosis seems more favorable. Specifically, tolerance is usually achieved by one year of age [104].

## 11. Unmet Needs and Conclusions

Fish allergy represents a relevant illness in pediatric age, with a worldwide prevalence stated between 0% and 7%. In most cases, the reactions are IgE-mediated and can occur after ingestion, skin contact, or inhalation of the antigen. Non-IgE-mediated fish allergy, such as FPIES, has also been reported. Clinically, three clusters have been defined: polysensitized patients to all types of fish, mono-sensitized patients reactive to one species, and oligo-sensitized patients reacting to some specific fish. Parvalbumin is the main fish allergen responsible for allergic reactions in most patients. Skin prick tests and specific IgE are the most used tests. However, oral challenge remains the diagnostic gold standard for fish allergy in children. Clinical management of fish allergy remains the food elimination diet. Since most patients with fish allergies can eat some species of fish, it is important to prevent unnecessary elimination diets in light of the numerous nutritional benefits of this food (e.g., enriching omega-3 fatty acids and valuable protein content). Recent studies have examined molecular diagnostics and BAT as new tools for predicting clinical reactivity, reducing the need for oral challenges and helping with more specific advice on restrictive diets. Comparative studies would be beneficial on the clinical cross-reactivity to different fish species in the pediatric population with the aim of improving management of fish allergy. Broadening the molecular knowledge of fish allergens allowed the development of hypoallergenic recombinant parvalbumins with preserved immunogenicity for advancing specific immunotherapy, such as desensitization to fish. This would be an important basis for safer novel therapeutic approaches against fish allergy. These new promising therapeutic approaches are being studied to modulate the immune response in fish allergy.

## Figures and Tables

**Table 1:** Frequency of fish as offending food in children affected by food protein-induced enterocolitis (FPIES).

Author, Year	Country	Fish as Offending Food (%)	N	Age, Range–Mean
Maciag, 2020 [6]	Australia, Austria, Brazil, Egypt, Germany, Italy, UK, USA, Qatar	9.3	441	1.25–4 y (2 y)
Mehr, 2017 [7]	Australia	5	230	7–14 m (10 m)
Ruiz-Garcìa, 2017 [8]	Spain	31	16	11 m–12 y (50.6 m)
Douros, 2019 [9]	Greece	54	78	10.1–12.2 m (11.1)
Xepapadaki, 2019 [10]	Greece	34.7	72	12.1–19.5 m (15.8)
Miceli Sopo, 2012 [11]	Italy	12	35	10.6 ± 6.7 m
Miceli Sopo, 2015 [12]	Italy	81	70	6–46 m (14)
Vila, 2015 [13]	Spain	80.9	21	9 m–14 y (12 m)
Papadopoulou, 2020 [14]	Greece	56	100	13 ± 6.7 m
Vazquez-Ortiz, 2017 [15]	Spain	54.3	81	9–12 m (10)
Alonso, 2019 [16]	Spain	37.5	8	4–24 m
Dìaz, 2019 [17]	Spain	32.5	120	0.5 m–12 y (11.2 m)

**Table 2:** Prevalence of fish allergy in the general pediatric population by diagnostic workup, geographic location, and age.

Diagnosis	Prevalence	Age (Years)	Country	Author, Year	Ref.
Self-reported	5%	1–4	Finland	Pyrhönen, 2009	[28]
	7%	1–6	Finland	Kajosaari, 1982	[29]
	0.0001%	0–2	Israel	Dalal, 2002	[30]
	2.8%	6–9	United Arab Emirates	Al-Hammadi, 2010	[31]
	3%	0–2	Norway	Eggesbø, 1999	[32]
	0.1%	0–18	USA	Sicherer, 2004	[33]
	0.3%–0.6%	0–2/>11	USA	Gupta, 2011	[34]
	13%	0–18	Singapore	Chiang, 2007	[35]
	2.29%	14–16	Philippines	Connett, 2012	[36]
	1.1%	0–5	Thailand	Laoaraya, 2012	[37]
	1.62%	2–6	Vietnam	Le, 2019	[38]
	0.3%	5–16	Ghana	Obeng, 2011	[39]
Clinical history + specific IgE + skin prick test	0.3%	0–18	Finland	Von Hertzen, 2006	[40]
	1.3%	13–18	UK	Pereira, 2005	[41]
	0.7%	5–18	France	Pénard--Morand, 2005	[42]
	0.21%	0–18	China	Chen, 2011	[43]
	17.8%	1–7	Spain	Crespo, 1995	[44]
Oral challenge	0%	0–80	Denmark	Osterballe, 2005	[45]
	0.0006%	6	UK	Venter, 2006	[46]
	0.0002%	6–9	Turkey	Orhan, 2009	[47]
	0.2%	1	Island	Kristinsdottir, H. 2011	[48]

**Table 3:** Molecular bony fish allergens recognized by the allergen nomenclature database WHO/IUIS (World Health Organization/International Union of Immunological Societies).

Order	Species	Molecular Allergen	Biochemical Name	Prevalence (%)	Molecular Weight (kDa)
*Clupeiformes*	Herring (*Clupea harengus*)	Clu h 1	parvalbumin	45	12
	Sardine (*Sardinops sagax*)	Sar sa 1	parvalbumin	80	12
*Cyprinoformes*	Carp (*Cyprinus carpio*)	Cyp c 1	parvalbumin	100	12
		Cyp c 2	enolase	17	47
	Grass carp (*Ctenopharyngodon idella*)	Cten i 1	parvalbumin	94	9
*Gadiformes*	Cod (*Gadus callarias*)	Gad c 1	parvalbumin	100	12
	Cod (*Gadus morhua*)	Gad m 1	parvalbumin	100	12
		Gad m 2	enolase	56	50
		Gad m 3	aldolase	37	40
*Perciformes*	Tuna (*Thunnus albacares*)	Thu a 1	parvalbumin	95	11
		Thu a 2	enolase	19	50
		Thu a 3	aldolase	13	40
	Barramundi (*Lates calcarifer*)	Lat c 1	parvalbumin	77–83	11.5
		Lat c 6	collagen	22	130–140
	Tilapia (*Oreochromis mossambicus*)	Ore m 4	tropomyosin	100	33
	Indo-pacific mackerel (*Rastrelliger kanagurta*)	Ras k 1	parvalbumin	83	11.3
	Mackerel (*Scomber scombrus*)	Sco s 1	parvalbumin	95	12
	Swordfish (*Xiphias gladius*)	Xip g 1	parvalbumin	71	11.5
*Pleuronectiformes*	Yellow diamond (*Lepidorhombus whiffiagonis*)	Lep w 1	parvalbumin	100	11.5
*Salmoniformes*	Keta Salmon (*Oncorhynchus keta*)	Onc k 5	vitegenin	nd	18
	Rainbow trout (*Oncorhynchus mykiss*)	Onc m 1	parvalbumin	95	12
	Salmon (*Salmo salar*)	Sal s 1	parvalbumin	49–64	12
		Sal s 2	enolase	24–34	50
		Sal s 3	aldolase	16–26	40
		Sal s 4	tropomyosin	13	37
		Sal s 6	collagen	22	130, 140
		Sal s 7	creatine kinase	14	43
		Sal s 8	triose-P isomerase	34	25
		Sal s 9	nd	nd	nd
*Siluriformes*	Pangasius (*Pangasianodon hypophthalmus*)	Pan h 1	parvalbumin	42	11
		Pan h 2	enolase	21	50
		Pan h 3	aldolase	21	40
		Pan h 4	tropomyosin	6–32	35
		Pan h 7	creatine kinase	10	43
		Pan h 8	triose-P isomerase	19	21
		Pan h 9	pyruvate kinase	6	65
		Pan h 10	lactate DH	13	34
		Pan h 11	glucose-6-P DH	8	60
		Pan h 13	glyceraldehyde-3-P DH	6	36

Prevalence is extracted from reference www.allergen.org/literature, accessed on 25 May 2023. Abbreviations: DH, dehydrogenase; P, phosphate; nd, not determined.

## Data Availability

Data sharing not applicable.
